# In the Shadow of Loss: Coping Strategies and Support Needs of Pediatric Nurses

**DOI:** 10.1192/j.eurpsy.2026.11808

**Published:** 2026-07-31

**Authors:** A. Zartaloudi, C. Lekas, D. Marinaki, M. Zerdila, I. Koutelekos

**Affiliations:** 1 Department of Nursing, Associate Professor, University of West Attica; 2 Nurse, MSc in General Pediatrics and Pediatric Subspecialties - Clinical Practice and Research; 3 Mental Health Nurse, MSc, “Sismanogleio -Amalia Fleming” General Hospital; 4 Nurse, MSc, “Georgios Gennimatas” General Hospital, Athens, Greece

## Abstract

**Introduction:**

Pediatric nurses are frequently confronted with the death of children, an experience that represents one of the most emotionally challenging aspects of their profession. Their attitudes, coping strategies, and perceived support needs are crucial in shaping both the quality of care provided and their own psychological well-being.

**Objectives:**

The present study aimed to explore pediatric nurses’ coping strategies, and perceptions of support needs in clinical practice.

**Methods:**

A cross-sectional descriptive design was applied. Data were collected using a structured questionnaire specifically designed for the purposes of this study, which was administered to one hundred and seventy pediatric nurses working in hospital settings.

**Results:**

The most common coping strategy reported by nurses was reflecting on children’s death in their home environment (43%), while 34% either avoided discussing the issue with colleagues or experienced social withdrawal. Only a small proportion reported crying as an outlet. A majority (62.8%) emphasized the importance of continuing family care during the mourning period, and 70.6% strongly supported participation in psychological support groups for nurses caring for dying pediatric patients. Nurses who felt well prepared to address death expressed less fear of it.

**Conclusions:**

Pediatric nurses adopt diverse coping strategies when faced with the death of children, yet many express a strong need for structured psychological support. Educational preparation and participation in support groups appear to mitigate fear of death and promote healthier coping. Interventions addressing emotional resilience and professional preparedness are essential for sustaining both nurses’ well-being and the quality of pediatric end-of-life care.

**Disclosure of Interest:**

None Declared

